# Transmission of Ranavirus between Ectothermic Vertebrate Hosts

**DOI:** 10.1371/journal.pone.0092476

**Published:** 2014-03-25

**Authors:** Roberto Brenes, Matthew J. Gray, Thomas B. Waltzek, Rebecca P. Wilkes, Debra L. Miller

**Affiliations:** 1 Department of Biology, Carroll University, Waukesha, Wisconsin, United States of America; 2 Center for Wildlife Health, University of Tennessee, Knoxville, Tennessee, United States of America; 3 College of Veterinary Medicine, University of Florida, Gainesville, Florida, United States of America; 4 College of Veterinary Medicine, University of Tennessee, Knoxville, Tennessee, United States of America; Institut Pasteur, France

## Abstract

Transmission is an essential process that contributes to the survival of pathogens. Ranaviruses are known to infect different classes of lower vertebrates including amphibians, fishes and reptiles. Differences in the likelihood of infection among ectothermic vertebrate hosts could explain the successful yearlong persistence of ranaviruses in aquatic environments. The goal of this study was to determine if transmission of a Frog Virus 3 (FV3)-like ranavirus was possible among three species from different ectothermic vertebrate classes: Cope’s gray treefrog (*Hyla chrysoscelis*) larvae, mosquito fish (*Gambusia affinis*), and red-eared slider (*Trachemys scripta elegans*). We housed individuals previously exposed to the FV3-like ranavirus with naïve (unexposed) individuals in containers divided by plastic mesh screen to permit water flow between subjects. Our results showed that infected gray treefrog larvae were capable of transmitting ranavirus to naïve larval conspecifics and turtles (60% and 30% infection, respectively), but not to fish. Also, infected turtles and fish transmitted ranavirus to 50% and 10% of the naïve gray treefrog larvae, respectively. Nearly all infected amphibians experienced mortality, whereas infected turtles and fish did not die. Our results demonstrate that ranavirus can be transmitted through water among ectothermic vertebrate classes, which has not been reported previously. Moreover, fish and reptiles might serve as reservoirs for ranavirus given their ability to live with subclinical infections. Subclinical infections of ranavirus in fish and aquatic turtles could contribute to the pathogen’s persistence, especially when highly susceptible hosts like amphibians are absent as a result of seasonal fluctuations in relative abundance.

## Introduction

The persistence of an infectious disease in the environment is directly related to the availability of suitable hosts and the likelihood of pathogen transmission. In aquatic environments, pathogens can be transmitted between hosts via direct contact, ingestion of infected tissue (e.g., predation), or by indirect waterborne contact [1], [2]. The route and magnitude of transmission depends on host density and environmental factors such as water temperature and pH [1]–[3]. When host densities are high, direct transmission via close contact, such as bumping or fighting, can occur. Conversely, when host densities are low or fluctuating in aquatic environments, indirect transmission through water may be most efficient [2], [3]. Many pathogens that inhabit environments with fluctuating host densities are able to infect several host species that have different levels of susceptibility, with some individuals maintaining subclinical infections thereby contributing to the persistence of the pathogen [1], [4].

Ranaviruses are large DNA viruses in the Iridoviridae family, a diverse group of viruses known to infect lower vertebrate hosts including amphibians [5]–[9], fish [10]–[13], and reptiles [14]–[17]. High variation in susceptibility of amphibians, fish and chelonians to ranaviruses has been reported [8], [12], [13], [18]–[20]. Differences in host susceptibility to ranaviruses create an ideal scenario for the pathogen to move between hosts, utilizing highly susceptible species for amplification and low susceptible hosts for persistence [21]. Reservoirs composed of subclinically infected hosts might explain the yearlong persistence of ranaviruses in the environment [22]. Many aquatic communities where ranaviruses emerge are composed of multiple species from different ectothermic vertebrate classes [23].

Given the variability in susceptibility to ranavirus for host species within and among ectothermic vertebrate classes, presumably one class could function as a reservoir for another class. In experiments that we performed [24], we demonstrated that interclass transmission was possible via exposure to ranavirus inoculum in water. Although water bath exposure to a standard concentration of ranavirus is useful to understand basic transmission mechanisms, it does not connote natural transmission between hosts. Thus, our goal was to test whether interclass transmission of ranavirus was possible between ectothermic vertebrate classes using previously exposed hosts as the source of the virus. These results are fundamental to understanding the epizootiology of ranaviruses.

## Methods

To determine the occurrence of ranavirus transmission between ectothermic vertebrate classes (fish, reptiles and amphibians), we set up experimental challenges between three sympatric species known to be susceptible to ranavirus infection: mosquito fish (*Gambusia affinis*; [24]), red-eared slider turtle (*Trachemys scripta elegans* [25]), and Cope’s gray treefrog (*Hyla chryscocelis* [9]). We used a Frog Virus 3 (FV3)-like ranavirus that was isolated from a pallid sturgeon (*Scaphirhynchus albus*) during a die-off in an aquaculture facility [26]. From previous single species challenges, the species we used were known to be susceptible to this isolate, exhibiting 85%, 35% and 5% mortality in Cope’s gray tree frog tadpoles [MJG, TBW, and DLM, unpubl. data], red-eared slider hatchlings [MJG, TBW, and DLM, unpubl. data], and mosquito fish [24], respectively, when exposed to the virus in water.

The mosquito fish used for the experiment were obtained as fingerlings (ca. 5–10 cm length) from a commercial hatchery and acclimated in the laboratory prior to the experiment for a week in a 1200-L tank with constant, flow-through water (75.7 L/s) that was dechlorinated and maintained at 25°C. Turtles were obtained as hatchlings (ca. 5 cm) from a commercial retailer (Turtle Shack, Port Richey, FL) and acclimated in a 1200-L tub for a week with floating platforms and lights for thermal and UV exposure (Zoo Med Powersun UV Self-Ballasted Mercury Vapor UVB Lamp, San Luis Obispo, CA). During the acclimation period, fishes and turtles were fed daily a commercial high protein fish food (Purina Mills Aquamax Pond Fish 4000 Catfish Food Pellets, Gray Summit, MO) *ad libitum*. Amphibian larvae were collected as egg masses from local wetlands, hatched and raised in 324-L wading pools. Tadpole acclimation, maintenance, and feeding protocols were identical to Hoverman et al. [9]. Upon purchase and arrival to the University of Tennessee, a random sample of five individuals of every species was humanely euthanized by immersion in benzocaine hydrochloride (100 mg/L; [27]), and tested for ranavirus infection using real-time quantitative PCR (qPCR; see methods below); all qPCR results were negative.

To test interclass transmission of the pathogen, we paired one virus-exposed individual with one unexposed individual of a different species. Virus-exposed individuals were exposed to 10^3^ plaque forming units (PFU)/mL of ranavirus in water [9], while unexposed individuals were exposed to the same volume of virus-free media (i.e., minimum essential media, MEM Eagle Sigma-Aldrich, Seelze, Germany). Exposure occurred individually in 2-L containers filled with 1-L of dechlorinated water for 3 d [9]. Thereafter, individuals were randomly selected and paired in the following arrangement: 1) exposed turtle and unexposed tadpole, 2) exposed tadpole and unexposed turtle, 3) exposed fish and unexposed tadpole, 4) exposed tadpole and unexposed fish, and 5) exposed tadpole and unexposed tadpole. An identical complement of paired control treatments (i.e., both species unexposed) also was included. Individuals were paired in 15.5-L containers divided in half by a 2000-μm plastic mesh (design adapted from Harp and Petranka [28])The tubs were placed on shelving units (122×244-cm) at two heights in a randomized block design, with shelf height as the blocking variable. There were 20 experimental units per treatment. Room temperature in the laboratory was maintained at 25°C and the photoperiod was set at 12∶12 day:night to emulate natural conditions [29].

After the inoculation period and every three days thereafter, water was changed (100% of volume) to maintain water quality during the experiment [9]. Amphibian larvae were fed ground fish food (TetraMin, Blacksburg, VA) at a ratio of 12% of their body mass after each water change [9]. Turtles and fish were fed high protein catfish pellets (Purina Mills Aquamax Pond Fish 4000 Catfish Food Pellets, Gray Summit, MO) every other day at a ratio of 3% of their body mass [30]. Five non-experimental individuals per species that were treated identical to controls were weighed for food ration estimates so not to introduce stress to experimental animals [8].

During the experiment, all individuals were monitored twice daily for morbidity. Individuals that exhibited morbidity consistent with ranaviral disease (i.e., petechial hemorrhages, edema, and loss of equilibrium) for >24 hours were humanely euthanized by double pithing [31]. Bodies of directly exposed individuals that were pithed were returned to the tub until the next water change to allow normal virus shedding post mortem. Conversely, unexposed individuals that were pithed were removed immediately from the containers. The duration of the experiment was four weeks (28 days), which is sufficient time to observe morbidity in ectothermic vertebrates exposed to ranavirus [8], [12], [32]. Any surviving individuals were humanely euthanized at the end of the experiment. Sections of liver and kidney were collected from all individuals for virus detection by qPCR. All husbandry and euthanasia procedures followed approved University of Tennessee IACUC protocol #2018.

We extracted genomic DNA (gDNA) from liver samples using the DNeasy Blood and Tissue Kit (Qiagen Inc., Valencia, CA). We used a Qubit fluorometer and the Quant-iT dsDNA BR Assay Kit to quantify the concentration of gDNA in each sample (Invitrogen Corp., Carlsbad, CA, USA). Our qPCR procedures and primers were identical to Picco et al. [33]. All extracted DNA samples were run in duplicate and an individual was declared infected if the qPCR cycle threshold (CT) was <30 for both samples. The CT was determined for our PCR system (ABI 7900Fast Real-Time PCR System; Life Technologies Corporation, Carlsbad, CA) by developing a standard curve using known quantities of ranavirus. Four controls were included in each qPCR assay: DNA extracted from a ranavirus-positive tadpole, DNA extracted from a ranavirus-negative tadpole, DNA extracted from our ranavirus isolate, and DNA grade water.

We were interested in determining if transmission occurred between paired hosts of different vertebrate classes, and if so, did percent transmission differ depending on the host. We also were interested in whether individuals survived with subclinical infections. Thus, we performed a chi-square test of homogeneity to test for the differences in infection prevalence and mortality for the unexposed individuals among the paired-host treatments. All analyses were performed using SAS 9.3 [34] at α = 0.05.

## Results

**Figure 1:**
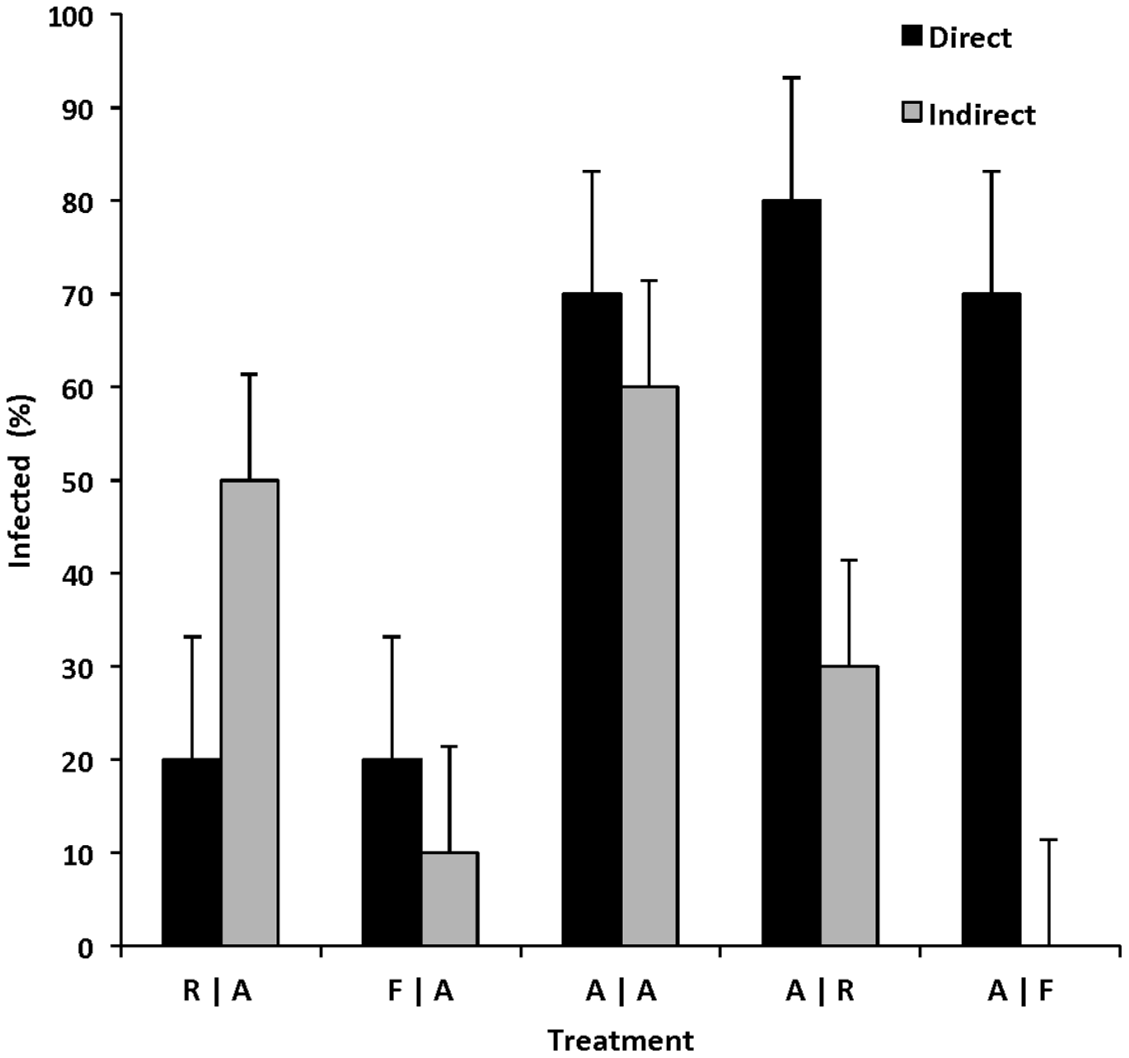
Infection of individuals exposed to ranavirus directly or indirectly. Infection prevalence between individuals exposed to ranavirus inoculum (direct) or via shedding (indirect) by a paired host. Treatments were paired individuals (*n* = 20 per bar) of different ectothermic vertebrate classes (A = amphibian, R = reptile, F = fish); thus, A|F = amphibian paired with fish. Infection of indirectly exposed individuals is evidence of waterborne transmission by directly exposed individuals.

**Figure 2:**
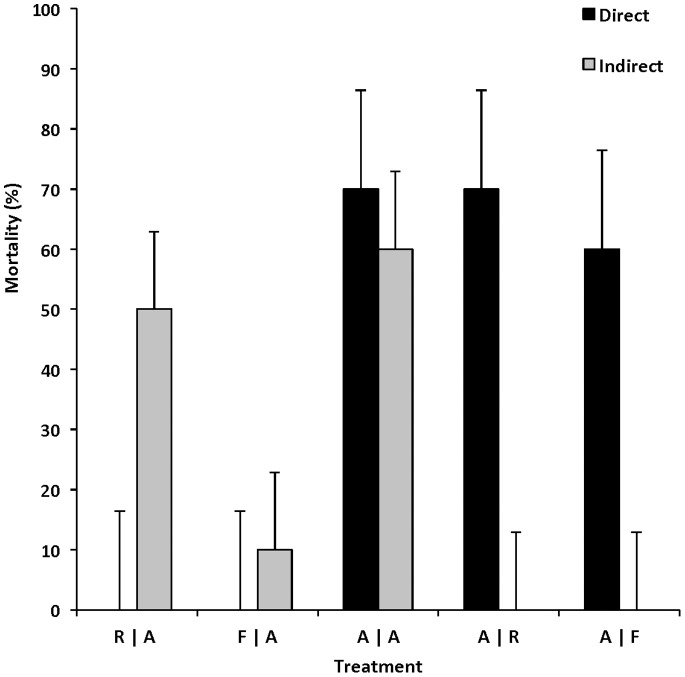
Mortality rate of individuals exposed to ranavirus directly or indirectly. Mortality between individuals exposed to ranavirus inoculum (direct) or via shedding (indirect) by a paired host. Treatments were paired individuals (*n* = 20 per bar) of different ectothermic vertebrate classes (A = amphibian, R = reptile, F = fish); thus, A|F = amphibian paired with fish.

We found that ranavirus could be transmitted between different vertebrate classes, and that infection prevalence (χ^2^_4_ = 24.76, *P<*0.001) and mortality (χ^2^_4_ = 36.40, *P<*0.001) differed among unexposed individuals depending on the paired species (Figure 1). Susceptibility of directly (i.e., inoculum exposed) and indirectly (shed by co-inhabitant) exposed individuals varied among host species. Amphibian larvae were the most susceptible vertebrate class with an average infection of 73% for individuals directly exposed to ranavirus, and 40% average infection for individuals exposed indirectly by other infected hosts (Figure 1). Ranavirus transmission from infected amphibian larvae to naïve hosts was observed in 60% of conspecifics and 30% of turtles, resulting in 100% and 0% mortality, respectively (Figure 2). No fish were infected or died after 28 days, despite being housed with a large percentage (70%) of infected amphibian larvae (Figures 1–2).

After 28 days, 20% of directly exposed turtles were infected while 50% of amphibians that were housed with them became infected and died (Figures 1 and 2), suggesting that at least 30% of turtles cleared the virus before the end of the experiment. Directly exposed fish showed low susceptibility to ranavirus (20% infection, 0% mortality). These individuals transmitted the virus to 10% of the co-inhabitant amphibian larvae – all of which died. No mortality occurred in our control treatments.

## Discussion

The objective of our study was to determine if ranavirus could be naturally transmitted between ectothermic vertebrate classes, which had not been demonstrated previously. Virus-exposed mosquito fish and red-eared sliders were able to transmit ranavirus to Cope’s gray treefrog tadpoles and cause 10% and 50% mortality, respectively. Exposed gray treefrog tadpoles were able to transmit ranavirus to red-eared sliders (30% infection), but none of the turtles died after 28 days. Exposed gray treefrog tadpoles were unable to transmit ranavirus to mosquito fish. Alternatively, mosquito fish may have become infected when exposed to infectious tadpoles but cleared the virus within 28 days when the experiment ended and surviving individuals were euthanized and tested for infection. Exposed gray treefrogs efficiently transmitted ranavirus to conspecifics (60% infection); all infected conspecifics died.

These results demonstrate that interclass transmission of ranavirus is possible through water by virion shedding from an infected individual. Previous studies [24], [35] have inferred interclass transmission by exposing a host in one vertebrate class to a ranavirus isolated from a different vertebrate class. Our results also suggest that larval amphibians might be amplification hosts as demonstrated by high infection prevalence and mortality; whereas, fish and aquatic turtles may function as reservoir species due to lower susceptibility [21]. Our experiment was conducted with only one species from each ectothermic vertebrate class. Experiments are needed with additional species to determine if this trend holds.

Our study was conducted with an FV3-like ranavirus initially isolated from pallid sturgeon. Several studies have demonstrated that different FV3-like isolates have different pathogenity [8], [36], and presumably transmission dynamics. Additionally, the role of fish, amphibians and reptiles as reservoirs or amplification hosts may be dependent on ranavirus species. For example, *Ambystoma tigrinum* virus (ATV) causes minimal infection and disease in anurans and fish [6], [37]. Thus, although our results demonstrate that our isolate can be transmitted among ectothermic vertebrate classes and amphibians appear to function as amplification hosts, more studies are needed with other isolates before broad inferences can be made on the role of vertebrate classes in the persistence and emergence of ranaviruses.

Levels of mortality observed in our study were slightly lower than individual species challenge studies performed by others. For example, Hoverman et al. [9] reported 80% mortality of Cope’s gray treefrog tadpoles exposed to an FV3-like ranavirus inoculum in a water bath. We found that 10% of mosquito fish became infected with half of those individuals dying when exposed to the same virus [24]. MJG, DLM, and TBW (unpubl. data) found that 35% of red-eared sliders became infected and died when exposed to the same isolate used in our experiment. The differences in mortality rates may be a consequence of virion concentration in the water. The aforementioned studies exposed hosts to 10^3^ PFU/mL of ranavirus, while unexposed individuals in our study were exposed to one individual that was previously exposed to ranavirus inoculum at 10^3^ PFU/mL. Individuals exposed to the inoculum may not have become infected or perhaps shed virions at a concentration <10^3^ PFU/mL when housed with a naïve individual. Dose dependency of ranavirus pathogenicity has been reported [38], [39]. Also, within a species, direct exposure of ranavirus inoculum resulted in greater infection and mortality than indirect exposure of ranavirus shed from a paired individual. These results suggest that challenge experiments that use ranavirus inoculum at 10^3^ PFU/mL may overestimate waterborne transmission dynamics among hosts.

Our study was conducted under controlled laboratory conditions; thus, may not represent true transmission dynamics in the wild. For example, the unsterile conditions of pond water and differences in temperature can affect the viability of ranavirus outside the host [40]. Other factors in the wild such as natural and anthropogenic stressors, direct contact of individuals, and necrophagy or predation can facilitate transmission of ranavirus [22]. To improve our understanding of interclass transmission dynamics in the wild, we recommend that future studies use outdoor aquatic mesocosms similar to Brenes [36] and Reeve et al. [41]. We also acknowledge that transmission dynamics during our study were followed for only 28 days. Longer duration studies will be useful in understanding the functional role of ranavirus hosts throughout the annual cycle.

Although transmission of ranavirus from an infected to naïve amphibian larvae via water bath has been documented previously [28], our results represent the first observation of high level of infection (60%) and mortality (50%) of amphibian larvae exposed to the pathogen solely by cohabitation with infected hosts. Harp and Petranka [28] reported low levels of infection (25%) and no mortality of naïve larval wood frogs (*Lithobates sylvaticus*) after 111 hours of cohabitation with infected conspecifics, attributing the low infection and lack of mortality to low levels of viral load shed by the moribund tadpoles. Although duration of cohabitation was longer in our study, Robert et al. [42] demonstrated transmission of ranavirus could occur as quickly as 3 hours in a water bath.

The capacity of subclinically infected fish and aquatic turtles to transmit ranavirus to amphibians has important implications regarding the persistence of this pathogen in aquatic environments. Reports of ranavirus outbreaks, particularly affecting amphibian communities, have been well documented [43]–[48]. In many cases, these outbreaks have been reported to be seasonally recurrent [44], [49], [50]. Most reports of recurrent ranavirus outbreaks in amphibian communities describe high levels of disease and mortality when amphibian larvae are highly abundant followed by a significant decrease of disease as the abundance of amphibian larvae decreases [51]. During these periods when amphibian density is low or completely absent from the water bodies, ranavirus appears to be absent, but ranavirus prevalence can increase rapidly as soon as the next generation of amphibians returns to the aquatic ecosystems [44], [49], [50]. It has been hypothesized that ranavirus can persist in aquatic environments via biological reservoirs [52]. Considering that interclass transmission is possible, ranaviruses might persist in fish and aquatic turtles when the availability of highly susceptible hosts like amphibian larvae is reduced [1], [39], [53].

According to Cronin et al. [54], ideal reservoir species are those that can harbor subclinical infections of pathogens without suffering impairment in their biological or ecological functions until the right conditions arrive and the pathogen can be released into the environment where it can invade new hosts more suitable for its replication. For this to occur, the pathogen must exhibit three basic characteristics [54]–[56]. First, it should display different levels of infectivity among hosts either by being able to infect different species at different rates or by infecting different ages or developmental stages of the same species at different rates. In the case of ranaviruses, differences in susceptibility ranging from low to high susceptibility has been described for amphibians species [8], [9] and life stages [57] as well as for fish [11], [12], [58] and reptiles species [16], [20], [32], [59]. Second, the pathogen must be able to be transmitted efficiently among hosts. Ranaviruses can be transmitted among hosts by contact [2], consumption (predation or cannibalism; [2]), and via water exposure [40], [60], [61]. Third, host availability should fluctuate through time between high and low susceptible species. Because of the complex life cycle and breeding phenology of amphibians, fluctuations in abundance and composition of amphibian communities is common [62], [63].

These three characteristics of the ranavirus-host system might facilitate the pathogen’s persistence. We hypothesize that ranaviruses persist at low prevalence in low susceptible hosts, such as aquatic turtles and fish, and emerge when highly susceptible hosts, such as many species of larval amphibians, become abundant. Thus, aquatic turtles and fish may function as reservoirs for ranavirus, while amphibian larvae may function as amplification hosts [52]. Moreover, if low susceptible hosts are highly mobile, they may contribute to overland transport of ranaviruses.

More research is needed on the susceptibility of other ectothermic vertebrates, especially turtles and fish, to understand the complex dynamics of ranaviruses in the environment throughout the year. Identification of amplification and reservoirs species will facilitate modeling of ranavirus transmission dynamics, and development of tools that could predict likelihood of ranavirus outbreaks. Knowledge of potential ranavirus reservoirs also could assist formulation of conservation strategies for areas where outbreaks have been documented. For example, removal of a fish reservoir might be a disease intervention strategy.
